# The impact of lesion vascularisation on tumours detection by electrical impedance scanning at 200 Hz

**DOI:** 10.2349/biij.3.4.e33

**Published:** 2007-10-01

**Authors:** A Malich, B Scholz, A Kott, M Facius, DR Fischer, MG Freesmeyer

**Affiliations:** 1 Institute of Diagnostic Radiology; Suedharz-Hospital, Nordhausen, Germany; 2 Siemens AG, Forchheim, Germany; 3 Institute of Diagnostic and Interventional Radiology, Friedrich-Schiller University Jena, Jena, Germany; 4 Institute of Diagnostic and Interventional Radiology, Inselspital Bern, Bern, Switzerland; 5 Institute of Nuclear Medicine, Martin-Luther-University Halle, Halle/Saale, Germany

**Keywords:** Breast cancer, electrical bio-impedance, diagnostic modality, measurement in vivo, biopsy-proven

## Abstract

**Objective:**

Cancer cells exhibit altered local dielectric properties compared to normal cells. These properties are measurable as a difference in electrical conductance using electrical impedance scanning (EIS). EIS is at present not sufficiently accurate for clinical routine despite its technological advantages. To modify the technology and increase its accuracy, the factors that influence precision need to be analysed and identified. While size, depth, localisation and invasiveness affect sensitivity, vascularisation might show an increased conductance and thus might affect specificity.

**Subjects and Methods:**

All patients were investigated with EIS (TransScan TS 2000, Migdal Ha Emek, Israel) Planned DCE-MRI prior to histological clarification were included (295 lesions). Dynamic enhancements were assigned scores after analysis of subtracted images after application of Gd-DTPA. D1: strong enhancement of >100% from initial signal obtained on native T1weighted sequence; D2: moderate enhancement 50-100%; D3: enhancement similar to glandular tissue, <50%; D4: subtle or no enhancement, less then surrounding glandular tissue.

**Results:**

89/113 malignant and 107/182 benign findings were visible by a focal increased conductance and/or capacitance using EIS (Sensitivity 79%, Specificity 59%). DCE-MRI was aborted due to claustrophobia in 17/295 cases. MR was used and out of 278 completed MR examinations, 101/104 malignant and 141/174 benign lesions were correctly diagnosed as benign or malignant leading to a sensitivity of 97% and a specificity of 81%. D1 benign lesions were positive in EIS in 33/55 cases suggesting a specificity of 44.4%. This value increases significantly with decreased vascularity to 68.9% (D2-4; 82/119). Out of 60 fibroadenomatous lesions, 10/23 fibroadenomas in class 1 had no focal increased conductance or capacitance and were thus considered as non-suspicious in EIS. The same result was applicable for the 29/37 benign lesions with a D2-4 contrast uptake (43.5% vs. 78.4%, p<.01).

**Conclusion:**

Vascularisation influences the measurable conductance at low frequency and therefore partially causes the insufficiently low specificity of EIS. Impedance measurements at frequencies in a range of 0.1 KHz to 1 MHz are required . According to theoretical and in vitro studies this might increase the accuracy of EIS technology. © 2007 Biomedical Imaging and Intervention Journal. All rights reserved.

## INTRODUCTION

The discrepancy in electrical capacitance of different types of tissue was first reported in the 1920s, reflecting the varying tissue characteristics of malignant tissue [1]. In the normal breast, moderate variations in impedance values are observed, expressing the differences among various types of breast tissue [1]. In contrast to these observations in normal tissue, malignant tumours show substantially increased capacitance and conductivity values resulting in decreased impedance [2, 3]. These discrepancies are attributed to changes in cellular water content, amount of extracellular fluid, packing density, destruction of tight junctions and cell membranes and a changed orientation of malignant cells [4]. Depending on the frequency used for calculations, the currently available technology TransScan TS 2000 (ISRAEL) allows a calculation mainly in the extracellular area by using a low frequency range.

Some studies report various sensitivity values even though EIS was used in a similar study design [6, 7, 8, 9, 10]. In order to improve the accuracy of this technology, the knowledge of influencing factors is required.

Therefore, recently published studies focused mainly on sensitivity influencing factors: size, depth, geometry and invasiveness that influence sensitivity are examples. In contrast to these studies, reasons for rather moderate specificity were mainly attributed to skin artefacts. It is questionable whether benign structures induce field imbalances (vortexes) by their bioelectrical properties. At low frequencies, the vessel walls could potentially act as insulators and thus would not contribute to conductance. As stated by Tofts et al., quantitative characterisation of the enhancement curves requires a complete understanding of the underlying physiological mechanisms, associated with the generation of the enhancement curve. As discussed by Tofts, the space into which Gd-DTPA can leak from the tumour capillaries probably includes the extracellular space. This is the key structure to be measured by low frequency impedance calculations.

Tofts’ studies proved that permeability of a tumour can be derived from the contrast uptake and from T1w-images. Permeability, however, influences transmittance which can be detected theoretically even at low frequencies.

According to Tofts’ analysis, highly vascularised lesions are characterised by a significantly increased permeability. Therefore a higher vascularisation might lead to an increased conductance because of the higher permeability of the cell membranes. The study analysed whether the vascularisation of lesions influenced the specificity or not.

## PATIENTS AND METHODS

A retrospective study design was used. All patients, who underwent electrical impedance scanning due to a suspicious finding in mammography and ultrasound, and who additionally had a DCE-MRI prior to histological clarification were included in the analysis.

In total 113 malignant and 182 benign findings were included.

### EIS principle and measurement

A low-level electrical voltage was applied to a metal cylinder that patients held in their hands while in a supine position. A cutaneous scan probe was placed at the region of interest exactly above the suspicious lesion. This was carried out under sonographic guidance. We used special ultrasound gel as recommended by TransScan® to accomplish a steady contact with an electrical current flowing through the patient’s body from the metal cylinder to the scan probe.

It can be assumed, that during passage through the breast, the electrical field and thus current distribution was quite homogeneous, because the healthy breast tissue was approximated as uniform. In contrast to healthy tissue, malignant lesions are characterised by a higher conductivity disturbance within the healthy tissue. In case of a disturbance of the electric field, i.e., due to the existence of a tumour, the electrical field will be disturbed as well. If this lesion is located next to the skin, the disturbance can be measured by a focal increased density of current and thus an increased focal conductance. The values of transmittance on the same electrode array at 200 Hz were interpolated, and finally displayed in real-time as a grey level impedance map. According to our own studies, lesions to a depth of 30mm can be detected. Simulations made by Scholz and coworkers demonstrated a detectability of lesions to a depth of 4 cm. Due to many influencing factors, absolute values of conductance and capacitance are less relevant because they vary significantly among different patients due to heterogeneity of skin impedance, different pressure of the hand held probe (varying contact), different skin moisture, different sizes of field disturbances, different depths of the lesions from the skin surface and different tissue composition of the examined breasts.

Absolute values of each electrode are therefore automatically calculated over a mean by the system. The resulting values are transferred into 256 different grey levels. If the value of one or several corresponding electrodes is higher than the mean of all electrodes, they are displayed as more luminous in the resulting image than the values obtained from the surrounding electrodes. Consequently such increased conductance values are visible as a lucent, enhancing spot. Examples of a focal enhanced structure and a non-enhanced calculation are given in Figure 2.

In our setup, analysis of EIS was performed immediately after the ultrasound examination. The position of the patient was unchanged. Therefore the radiologist was not ignorant of the mammographic and sonographic analysis. Ethical board approval was obtained. The technology used was approved by FDA.

**Figure 1:**
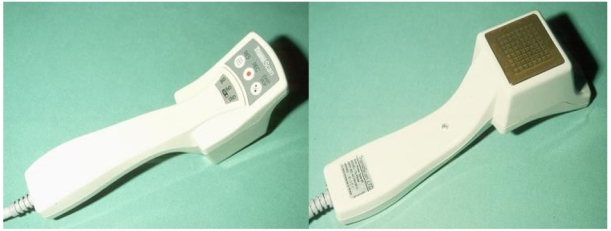
Image of both views of a probe of the EIS-technology

**Figure 2:**
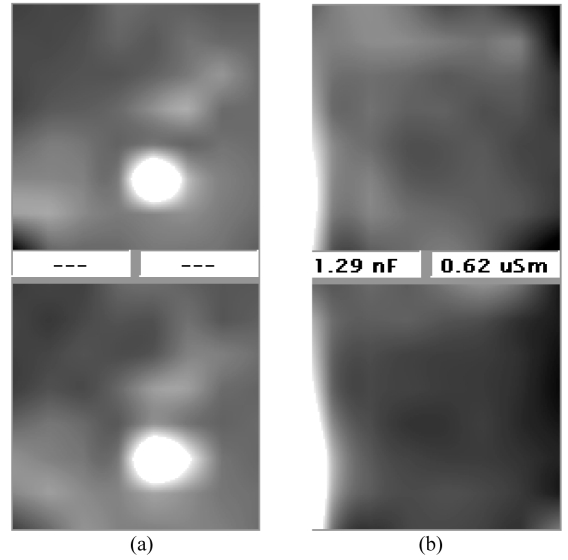
Example of a) a positive and b) a negative signal (upper quadrant capacitance, lower quadrant conductance) of an electrical impedance image.

The scan probe contains a planar array of 8x8 sensors. Each sensor is 3 mm x 3 mm in size. The centre to centre distance is about 4 mm, thus leaving a gap of 1 mm between adjacent electrodes (Figure 1). Good contact both on the probe and the metal cylinder is facilitated with the use of ultrasound gel. The sensor consists of a matrix of electrodes on the scan probe. It measures electrical currents (current distribution and indirectly voltage and resistivity applying frequencies ranging from 200-5000Hz). The only frequency used for analysis is 200 Hz. Examples of EIS calculations are given in Figure 2.

One examination lasted approximately 5 minutes.

Skin lesions, scars, moles, contact artefacts, bone, or air bubbles can induce spot-like results and thus influence the specificity.

### MR procedure

MR was performed after mammography, ultrasound and EIS by a different radiologist, who was not meant to be unaware of the previous mammography and ultrasound results but was also not informed regarding the EIS. A predefined MR examination-protocol was used and applied for all patients. All MR images were obtained with a 1.5 Tesla machine using a double-breast coil, with the patient in a prone position.

Multislice 2D Flash-T1-weighted images served as a sequence for the dynamic study. After acquisition of precontrast images, Gd-DTPA (Magnevist, Schering, Germany) was administered intravenously (0.1 mmol/kg) as a rapid bolus within 10 seconds followed by 20 ml saline flush. 35 seconds after bolus injection and saline administration, dynamic scanning was continued in the same sequence and under identical tuning conditions at 1-minute intervals for a total of 8 minutes. Precontrast T1 weighted images of the dynamic study were subtracted from the postcontrast dynamic T1 weighted images. The vascularisation was scored according to time-intensity curves. Therefore a region of interest (ROI) was placed on the area of the enhancing lesion with the most suspicious contrast uptake according to ACR-recommendations. The analysis was adapted to the ACR-criteria of enhancement and to the Fischer score used for differentiation of breast lesions in DCE-MRI. The density value obtained on the native T1-weighted scan was taken and subtracted from the density value obtained on exactly the same position on the T1 weighted image performed 1 minute after contrast uptake according to the recommendations of Kaiser et al. and ACR.

Lesion contrast uptake was scored as follows:

- D1: initial enhancement >100%
- D2: initial enhancement 50-100%,
- D3: initial enhancement <50%, uptake similar to the surrounding breast tissue.
- D4: subtle or no enhancement, lower then surrounding tissue.

Automated motion correction was not applied. Few cases had to be excluded due to severe motion artefacts on related irregular conditions to place the region of interest on exactly the same anatomical structure prior and after contrast application.

## RESULTS

### EIS overall performance parameters

89 of 113 histologically proven malignant lesions were detected by a focal increased conductance and/or capacitance.

107 of 182 benign lesions revealed a homogeneous conductance and capacitance (and thus no spot) using EIS. But 75 of the histologically proven benign lesions, showed a focal enhancing pattern similar to a malignant lesion and therefore were judged falsely positive.

In the classification of equivocal suspicious lesions, EIS achieved an overall sensitivity of 78.8% and a specificity of 58.8%. Negative and positive predictive values were 81.7% and 54.3%, respectively. Accuracy was calculated as 68.8%.

### MR overall performance parameters

17 out of the 295 examinations had to be aborted mainly due to claustrophobia and severe motion artefacts. Thus, MR-results are available for 278 lesions only. Using DCE-MRI 101/104 malignancies and 141/174 benign lesions were correctly detected after analysis of dynamic and morphologic features suggesting a sensitivity of 97.1% and a specificity of 81.0%. Consequently, positive and negative predictive values were 75.4% (calculated as 101/(101+33)) and 97.9% (141/(141+3)) respectively. Accuracy of DCE-MRI was 89.1% ((97.1%+81.0%)/2).

Mean size of benign and malignant findings did not differ significantly (sizes calculated using T1 weighted post-enhancement images: 18mm and 17mm in the mean, respectively).

### EIS-Performance of malignant lesions in relation to vascularity

Out of the 113 malignant lesions (including carcinomata in situ), 100 were classified as D1 and 4 lesions as D2-4. The remaining malignant lesions were not classifiable (reasons are, as given above claustrophobia, obesity). Out of the D1-lesions, a focal enhanced conductance was observed in 81 cases (81%). Of the four lesions classified as D2-4, one (D3) had the same focal enhanced conductance value.

### EIS-Performance of benign lesions in relation to vascularity

Of the 174 benign lesions with an MR-examination and an EIS-examination, 55 were classified as D1, of which EIS showed a positive focal increased conductance in 33 lesions, suggesting a specificity of 40.0%.

Among the 24 verified benign lesions of category D4, 6 were positive in EIS suggesting a specificity of 75.0%.

Comparing D1 versus all other lesions (D2+3+4), specificity differs significantly: 40.0% vs. 68.9% (22/55 and 82/119 lesions) according to Fisher’s t-test.

### EIS-Performance of fibroadenomatous changes in relation to its vascularity

The largest group of histologically verified lesions included fibroadenomas. Of these 60 lesions, 23 were classified as D1 of which 10/23 revealed a homogeneous field in EIS, resulting in a specificity of 43.5%. Of the other 37 lesions, 29 showed a homoegeous image in EIS, therefore suggesting a specificity of 78.4%.

Using Fisher’s t-test, the differences between D1 and D2-4 on fibroadenomatous tissue are significant (p<.01).

## DISCUSSION

General performance of Electrical Impedance Scanning compared to DCE-MRI

As proven in our study as well as in other studies, DCE-MRI is highly sensitive in the detection of invasive malignancies and reveals a high specificity. The major weaknesses of DCE-MRI are unavailability, length of procedure and monetary costs.

Hence electrical impedance scanning could be of interest as a cheap and fast technology to be used as an adjunctive examination in those cases where DCE-MRI is not available or where an MR-examination is contraindicated.

According to our experience, the sensitivity of EIS in its current application mode is definitely unable to achieve the impressive sensitivity (98%; 16) and specificity values documented for DCE-MRI, even if all currently known limitations are taken into account. In order to improve EIS, some modifications are necessary:

Vascularisation seems to influence the detection rate of EIS. Therefore benign lesions with a relevant disturbance of the electric field cause false positive conductance values. Due to the study design, the documented correlation between these two factors does not inevitably imply a cause-effect relationship. However, no other study has investigated this influence, so far. Furthermore the number of implemented histologically verified lesions with associated MR-examination allows a statistical analysis.

Consequently, the result of this study proposes, that the low specificity is not only caused by several mainly skin-associated artefacts, but also by some of the lesions being analysed. This result concurs with several in vitro studies.

It is necessary to detect benign lesions that induce changes in homogeneity in the electric field and thus result in a positive EIS-result. It is equally necessary to detect malignant and premalignant lesions that are not associated with a detectable inhomogeneity of electrical field. Furthermore, alternative factors that lead to poor specificity have to be seriously considered and embedded in future technological improvements. A few suggestions to enable developments in EIS will be discussed:

1. Jossinet and coworkers reported altered conductance and capacitance during different applied frequencies depending on the underlying histopathology. Therefore the analysis of the impedance in a range of frequencies including frequency-values of the beta-range could solve the problems of highly vascularised lesions, which include the wrongly detected benign lesions in EIS. Early prototypes have been built to analyze this in vivo in a frequency range of up to 1 MHz [25].
2. One further limitation of currently available EIS is that low frequency current does not pass cell membranes. This is the reason why intracellular changes cannot be obtained by EIS in the current available version. Jossinet and coworkers published results analysing impedivity of breast tissue over a frequency range [3, 5, 21]. As a result of their studies a divergence of the values within the range 10 kHz to 1 MHz can be apostrophised. Due to the fact that the conductivity-frequency relation is tissue characteristic [3, 21,22, 23], further information can be obtained by the calculated parameters including intracellular changes allowing the discrimination of malignant and benign lesions [3, 5, 24]. This explains the inability of EIS to detect non-invasive premalignant structures.
3. Additionally a high number of different artefacts, mainly associated with the skin surface (scars, hairs, bones, contact problems, etc.) reduce the specificity of EIS, as was demonstrated in this study. Therefore improvements should implement the option to determine the impedance values in various distances from the skin. By doing so the observer could match ultrasound performance in peak conductance and the depth of this peak-inducing lesion.. The proof of this principle was carried out in vitro as well as on clinical cases using post-processing algorithms (multisignal analyses) [17].
  Due to electrophysiological reasons, skin-associated alterations of conductance cannot induce artefacts in higher frequency-ranges to the same extend as in low frequencies. Taking those aspects into account, the high frequency analysis of lesions offers further diagnostic potential.
4. The EIS technique measures the current flow (interpolated by computers into changes of conductivity and capacitance). If the tumour size is large, the conductance of several electrodes on the scanner increases thus increasing the mean value of conductance over all sensors. Spot-like enhanced peaks cannot be expected in this case and a rather homogeneous brighter area will be displayed because the relative differences of conductance between closely located electrodes are smaller. This explains why larger, homogeneously-structured lesions do not induce a visible focal increase of electrical parameters and may eventually provide negative EIS results and thus a lowered sensitivity. Unfortunately, until now neither size nor depth information has been taken into account while comparing absolutely measured values with already histologically verified lesions.
5. The different vascularity of a breast lesion forms an input for low frequency impedance calculations and thus influences the EIS-result. Using MR, vascularisation of lesions can be obtained starting with (at least 3mm) due to the tumour neoangiogenesis being induced from this size on. It can be verified, that similar to MR, EIS (under optimal circumstances) allows tumour detection starting from this size [18]. The most obvious reason seems to be, that vascularisation / permeability of tumours and therefore the extracellular content is altered due to neoangiogenesis.

In contrast to other imaging modalities, MR detects a change in the signal after contrast application versus a pre-contrast value. It can be assumed, that vascular density, permeability as well as neovascularity are associated with the extent and character of this contrast uptake. However, more detailed analyses require pathological verifications which were completed when dynamic MR was introduced [19, 20]. It is widely accepted today, that contrast enhancement of breast lesions is an important diagnostic feature that reflects the angiogenesis of the tumour.

For the very first time, recently established, new CAD systems allow a clear identification of contrast uptake of the entire enhancing breast lesion. This new feature may reveal further options in the comparison of vascularisation, perfusion and impedance of breast lesions [26]. Tofts and coworkers proved, that permeability of a breast lesion, and thus the impedance, is closely related to the dynamic pattern obtainable in MRI [26].

## SUMMARY

Electrical impedance scanning shows promising potential for further evaluation of equivocal suspicious mammographic and/or ultrasound findings, especially as an adjunctive diagnostic method.

Vascularisation of lesions influence the low-frequency based calculation of conductance.

Technological developments are necessary to address factors that influence EIS performance. The analysis methodology of EIS has to be redefined to include depth and size-dependent analysis options and MUSIC-based calculations. A range of frequencies up to 1 MHz have to be implemented in the analysis.

Due to its high accuracy and sensitivity, DCE-MRI is the method of choice in the discrimination of equivocal and suspicious breast findings despite its limitations and rather high costs.
